# Overexpressing Carotenoid Biosynthetic Genes in *Synechocystis* sp. PCC 6803 Improved Intracellular Pigments and Antioxidant Activity, Which Can Decrease the Viability and Proliferation of Lung Cancer Cells In Vitro

**DOI:** 10.3390/ijms24119370

**Published:** 2023-05-27

**Authors:** Maturin Natesungnoen, Varisa Pongrakhananon, Peter Lindblad, Saowarath Jantaro

**Affiliations:** 1 Laboratory of Cyanobacterial Biotechnology, Department of Biochemistry, Faculty of Science, Chulalongkorn University, Bangkok 10330, Thailand; 2 Department of Pharmacology and Physiology, Faculty of Pharmaceutical Sciences, Chulalongkorn University, Bangkok 10330, Thailand; 3 Microbial Chemistry, Department of Chemistry—Ångström, Uppsala University, P.O. Box 523, SE-75120 Uppsala, Sweden

**Keywords:** carotenoids, *Synechocystis* sp. PCC 6803, lung cancer cells, antioxidant activity

## Abstract

In the antioxidant system in cyanobacteria, non-enzymatic antioxidants, such as carotenoids, are considered good candidates for coping with oxidative stress, particularly light stress, and pharmaceutical therapeutic applications. A significant amount of carotenoid accumulation has been recently improved by genetic engineering. In this study, to achieve higher carotenoid production with higher antioxidant activity, we successfully constructed five *Synechocystis* sp. PCC 6803 strains overexpressing (OX) native genes related to the carotenoids biosynthetic pathway, including OX_*CrtB*, OX_*CrtP*, OX_*CrtQ*, OX_*CrtO*, and OX_*CrtR*. All of the engineered strains maintained a significant quantity of myxoxanthophyll, while increasing zeaxanthin and echinenone accumulation. In addition, higher components of zeaxanthin and echinenone were noted in all OX strains, ranging from 14 to 19% and from 17 to 22%, respectively. It is worth noting that the enhanced echinenone component responded to low light conditions, while the increased β-carotene component contributed to a high light stress response. According to the higher antioxidant activity of all OX strains, the carotenoid extracts presented lower IC_50_ in lung cancer cell lines H460 and A549, with values less than 157 and 139 µg/mL, respectively, when compared with those of WTc, particularly OX_*CrtR* and OX_*CrtQ*. A higher proportion of zeaxanthin and β-carotene in OX_*CrtR* and OX_*CrtQ*, respectively, may considerably contribute to the ability to treat lung cancer cells with antiproliferative and cytotoxic effects.

## 1. Introduction

Carotenoids (Cars), present in all oxygenic photoautotrophic organisms, are intracellular pigments that are typically encased in cellular membranes and assist in the assembly and function of photosynthetic complexes [1,2,3,4,5]. Carotenes and their oxygenated derivatives, i.e., xanthophylls such as echinenone, myxoxanthophyll, nostoxanthine, and zeaxanthin, belong to a group of Cars. Their functions serve as not only accessory light-harvesting pigments but also as photoprotective agents when cells are exposed to light stress, such as high light intensity and ultraviolet radiation [6,7,8]. Regarding the hydrophobic molecules of β-carotene and echinenone, they are able to form complexes with proteins, thereby functioning as a bridge between proteins in photosynthetic systems [1,5], as well as a scavenger of singlet oxygen [9,10,11]. The xanthophylls were found to have a stronger scavenging character than the carotenes [12]. Although there is currently no evidence that the xanthophylls bind to membrane proteins, it was expected that xanthophylls would rigidify membranes, while β-carotene and echinenone probably have a fluidizing impact [2]. In photosynthesis, Cars can act as antioxidants, detoxifying harmful reactive oxygen species (ROS); accept the excess energy from photosensitized molecules such as chlorophyll or singlet oxygen; and form a triplet state before being released it as heat to the ground state [12]. In terms of human health and disease treatment, dietary Cars or foods rich in Cars are considered beneficial in preventing severe diseases such as cancer, psoriasis, cardiovascular diseases, neurodegenerative disorders, and ocular disorders [13,14,15,16]. Cars have a certain influence on diminishing the oncogenic progress by, for instance, controlling oxidative stress, apoptosis, cell cycle progression, and metastasis [16,17,18]. In addition, Cars serve as antioxidants in normal cells, while they act as pro-oxidants in cancer cells by interacting with lipid peroxyl radicals to form the Car radical cation (Cars•+), resulting in a higher ROS level with higher damage to cellular biomolecules [16,19,20].

**Figure 1:**
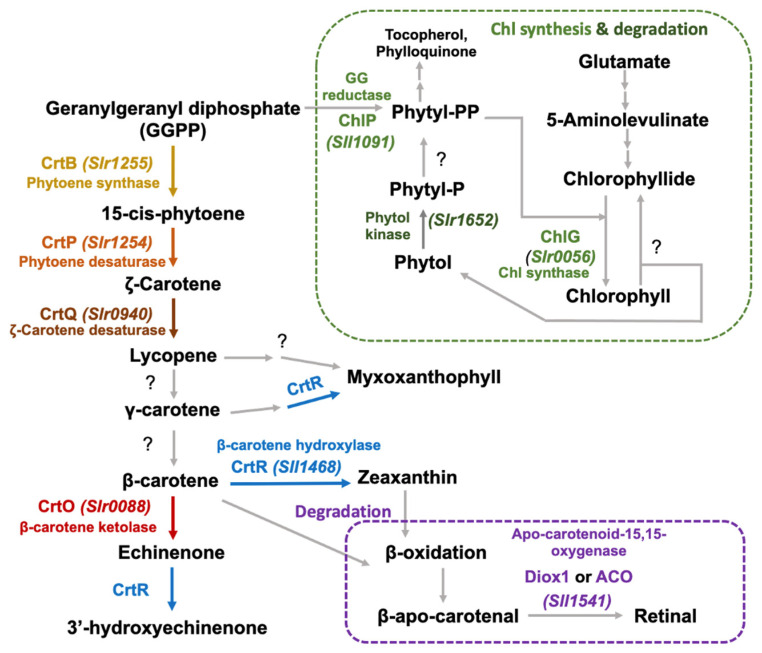
A tentative scheme of carotenoid synthesis and degradation in relation to chlorophyll synthesis and degradation (modified from [1,25,27]). Genes encoding enzymes in each pathway are indicated in italics. The scheme indicates “?” representing enzymes that are encoded by unidentified genes in *Synechocystis* sp. PCC 6803.

In Figure 1, the Car biosynthetic pathway in the cyanobacterium *Synechocystis* sp. PCC 6803 starts from the condensation of geranylgeranyl pyrophosphate (GGPP, 2 molecules) to generate 15-cis-phytoene via a phytoene synthase (CrtB, EC 2.5.1.32, encoded by the *slr1255* gene). Phytoene is then converted to respective ζ-carotene and lycopene via phytoene desaturase (CrtP, EC 1.3.5.5, encoded by the *slr1254* gene), and ζ-carotene desaturase (CrtQ, EC 1.3.5.6, encoded by *slr0940* gene). Although the genes encoding the lycopene ebsilon cyclase and lycopene beta cyclase (EC 5.5.1.19) responsible for the cyclization reaction of lycopene to γ-carotene and β-carotene, respectively, have been predicted in some cyanobacteria as CrtL-type, such as *P9211_07411*(ebsilon)/*P9211_11261*(beta) of *Prochlorococcus marinus* str. MIT 9211, *sync_0974*(beta) of *Synechococcus* sp. CC9311, and CruP and CruA of *Synechococcus* sp. PCC7002 [21], it has not yet been identified in *Synechocystis* strains in the Cyanobase database (https://genome.microbedb.jp, accessed on 28 April 2023). However, the *Sll0254* gene encoding bifunctional lycopene cyclase/dioxygenase (CrtL^diox^) in *Synechocystis* sp. PCC 6803 was previously considered to be required in myxoxanthophyll biosynthesis, but there has not yet been experimental evidence on its catalyzing β-carotene production [22]. Then, a β-carotene intermediate is converted to echinenone using the β-carotene ketolase (CrtO, EC 1.14.99.63, encoded by the *slr0088* gene). The localization levels of CrtQ and CrtO enzymes are abundantly located in plasma membranes, as compared to in thylakoid membranes [1]. Subsequently, β-carotene hydroxylase (CrtR, EC 1.14.13.129, encoded by the *sll1468* gene) effectively converts β-carotene to zeaxanthin, as well as myxoxanthophyll and 3′-hydroxylechinenone syntheses [1,23,24]. On the other hand, the connected biosynthetic pathways between carotenoids and chlorophyll occur at the GGPP intermediate which can be converted to phytyl-PP by geranylgeranyl reductase (ChlP, EC 1.3.1.83, encoded by the *sll1091* gene) [25]. In addition, chlorophyll synthase (ChlG, EC 2.5.1.62, encoded by the *slr0056* gene) catalyzed the last reaction of chlorophyll synthesis by esterifying chlorophyllide with phytyl-PP. For chlorophyll degradation, although there is still no gene encoding chlorophyllase retrieved in *Synechocystis* sp. PCC 6803, the phytol kinase (EC 2.7.1.182) encoded by the *slr1652* gene is found to catalyze the conversion of phytol to phytol monophosphate (phytol-P). Meanwhile, the degradation of carotenoids occurs via non-specific mechanisms, such as oxidation, and generates β-apo-carotenal, which is further converted to retinal using the Diox1 or ACO enzyme (EC 1.14.99.36, encoded by the *sll1541* gene) [26,27].

In this study, we improved total Cars and Car components with higher antioxidant activity by constructing five engineered *Synechocystis* sp. PCC6803 strains with overexpressing genes involved in the carotenoid biosynthetic pathway (Figure 1). Five engineered strains consisted of (1) *Synechocystis* sp. PCC6803, overexpressing the *slr1255* gene or OX_*CrtB*, (2) *Synechocystis* sp. PCC6803, overexpressing the *slr1254* gene or OX_*CrtP*, (3) *Synechocystis* sp. PCC6803, overexpressing the *slr0940* gene or OX_*CrtQ*, (4) *Synechocystis* sp. PCC6803, overexpressing the *slr0088* gene or OX_*CrtO*, and (5) *Synechocystis* sp. PCC6803, overexpressing the *sll1468* gene or OX_*CrtR*. In addition, all engineered stains contained a significant increase in total lipids when compared to the wild-type control. Particularly, the cell viability and proliferation of H460 and A549 lung cancer cell lines could be markedly reduced by carotenoid extracts from OX strains when compared to the wild-type control.

## 2. Results

### 2.1. Synechocystis sp. PCC 6803 Mutant Strains and Their Carotenoid Contents under Normal Growth Conditions

**Figure 2:**
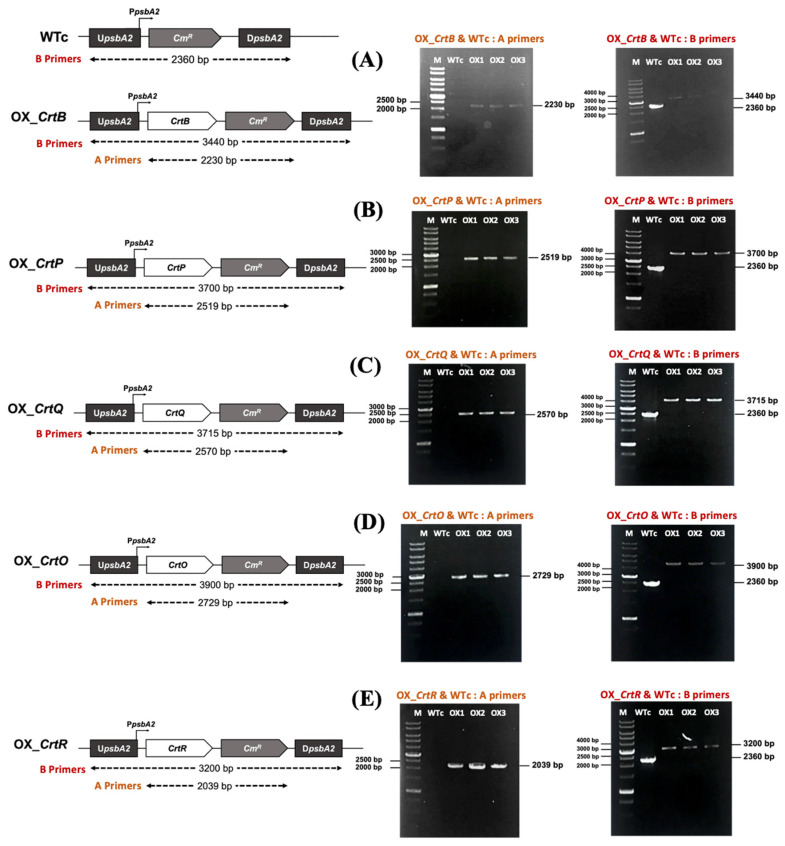
Genomic maps of engineered *Synechocystis* sp. PCC 6803 strains, including OX_*CrtB* (**A**), OX_*CrtP* (**B**), OX_*CrtQ* (**C**), OX_*CrtO* (**D**), and OX_*CrtR* (**E**). The specific primers (Supplementary information Table S1) were chosen to confirm the integration of each gene into the *Synechocystis* genome. All strains were constructed by overexpressing each native gene in the wild-type control. The confirmation of integration was verified by PCR with genomic DNA from WT and engineered strains as the template. The A Primers comprise a forward primer of a gene and a reverse primer of *Cm^R^*, while the B Primers are UppsbA2 and DSpsbA2 primers. Lane M: GeneRuler DNA ladder (Fermentas). For (**A**) for OX_*CrtB*, the A Primers were FW_*CrtB* and RV_*Cm^R^*; Lane 1: WTc, Lanes 2–4: clone numbers 1–3 (OX1, OX2, OX3). For (**B**) for OX_*CrtP*, the A Primers were FW_*CrtP* and RV_*Cm^R^*; Lane 1: WTc, Lanes 2–4: clone numbers 1–3 (OX1, OX2, and OX3). For (**C**) for OX_*CrtQ*, the A Primers were FW_*CrtQ* and RV_*Cm^R^*; Lane 1: WTc, Lanes 2–4: clone numbers 1–3 (OX1, OX2, and OX3). For (**D**) for OX_*CrtO*, the A Primers were FW_*CrtO* and RV_*Cm^R^*; Lane 1: WTc, Lanes 2–4: clone numbers 1–3 (OX1, OX2, and OX3). For (**E**) for OX_*CrtR*, the A Primers were FW_*CrtR* and RV_*Cm^R^*; Lane 1: WTc, Lanes 2–4: clone numbers 1–3 (OX1, OX2, and OX3).

Six engineered *Synechocystis* sp. PCC 6803 strains, including the wild-type control (WTc), OX_*CrtB*, OX_*CrtP*, OX_*CrtQ*, OX_*CrtO*, and OX_*CrtR* (Table 1), were constructed via a double homologous recombination. The wild-type control (WTc) strain was generated by substituting the *psbA2* gene with a *Cm^R^* cassette (Figure 2A), whereas each OX strain was created by substituting the *psbA2* gene with each gene fragment in the *Synechocystis* wild-type (WT) genome (Figure 2A–E). The complete segregation in *Synechocystis* genome was confirmed by PCR using UppsbA2 and DSpsbA2 primers (B Primers, Figure 2 and Supplementary Information Table S1). In the WTc strain, PCR products using B Primers showed no band, while it showed a 2.4 kb gene fragment when using A Primers. In Figure 2A–E, PCR products with the A Primers of OX_*CrtB*, OX_*CrtP*, OX_*CrtQ*, OX_*CrtO*, and OX_*CrtR* strains confirmed the correct sizes of 2.2, 2.5, 2.6, 2.7, and 2.0 kb, respectively, whereas PCR products with B Primers confirmed the correct sizes of 3.4, 3.7, 3.7, 3.9, and 3.2 kb, respectively.

**Figure 3:**
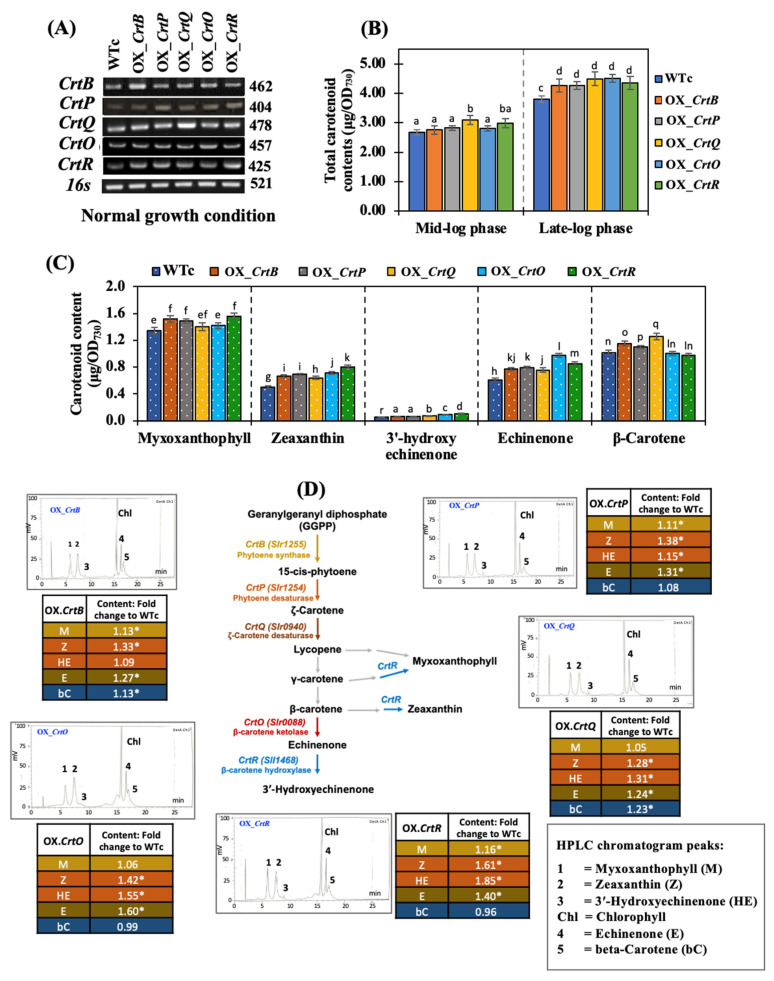
Transcript levels (**A**), total carotenoid contents (**B**), carotenoid contents (**C**), and fold changes of each content normalized with WTc and a HPLC chromatogram (**D**) of *Synechocystis* sp. PCC 6803 WTc and all engineered strains (OX). Cells were grown under normal growth conditions for 6 days (in (**B**)) and 12 days (**A**–**C**), representing the mid-log phase and late-log phases of cell growth, respectively. The error bars represent the standard deviations of means (mean ± S.D., n = 3). Means with the same letters have nonsignificant differences at a significant level of *p* < 0.05. The statistical difference of the data between the values of WTc and the engineered strain is indicated by an asterisk at * *p* < 0.05. For (**A**), all cropped gels were taken from the original images of RT-PCR products on agarose gels, as shown in Supplementary Information Figure S2.

In addition, RT-PCR data verified the gene overexpression in all OX strains with a higher transcript amount in comparison with those of WTc (Figure 3A). For total carotenoid content, the increased carotenoid accumulation in OX strains was noted when the cells were grown before reaching their late-log phase (12 days of cultivation) of growth under normal growth conditions (Figure 3B). At 12 days of growth, all OX strains cultivated under normal conditions had significantly increased contents of five Cars detected by HPLC, including myxoxanthophyll, zeaxanthin, 3′-hydroxy echinenone, echinenone, and β-carotene, with the exception of OX_*CrtQ*, OX_*CrtO*, and OX_*CrtR*, which had some reduced Car types (Figure 3C). The zeaxanthin and echinenone levels in all OX strains commonly increased by more than 1.2-fold as compared to WTc (Figure 3D). The results revealed that each component of the Car types was altered in the OX strains. OX_*CrtB* contained the increased amounts of all Car types, 1.1–1.3-fold that of WTc, except for 3′-hydroxyechinenone, whereas OX_*CrtP* contained the significant increases of all Car types, about 1.1–1.4-fold that of WTc, with the exception of β-carotene. For OX_*CrtQ*, the higher fold increase in each Car type was also observed, 1.2–1.3-fold that of WTc, except for myxoxanthophyll. Meanwhile, 3′-hydroxyechinenone and echinenone contents had a 1.6-fold increase compared with WTc, as noted in OX_*CrtO*. Interestingly, OX_*CrtR* had substantial increases in all Car types except for β-carotene; zeaxanthin levels increased by 1.6-fold; and the contents of 3′-hydroxyechinenone and echinenone increased by 1.9- and 1.4-folds, respectively. Although we did not monitor protein levels or enzymatic activity, all OX strains notably showed the altered changes in each Car product in accordance with their biosynthetic pathway.

### 2.2. Growth and Intracellular Pigment Levels of Chlorophyll a and Carotenoids under Various Light Intensity Conditions

**Figure 4:**
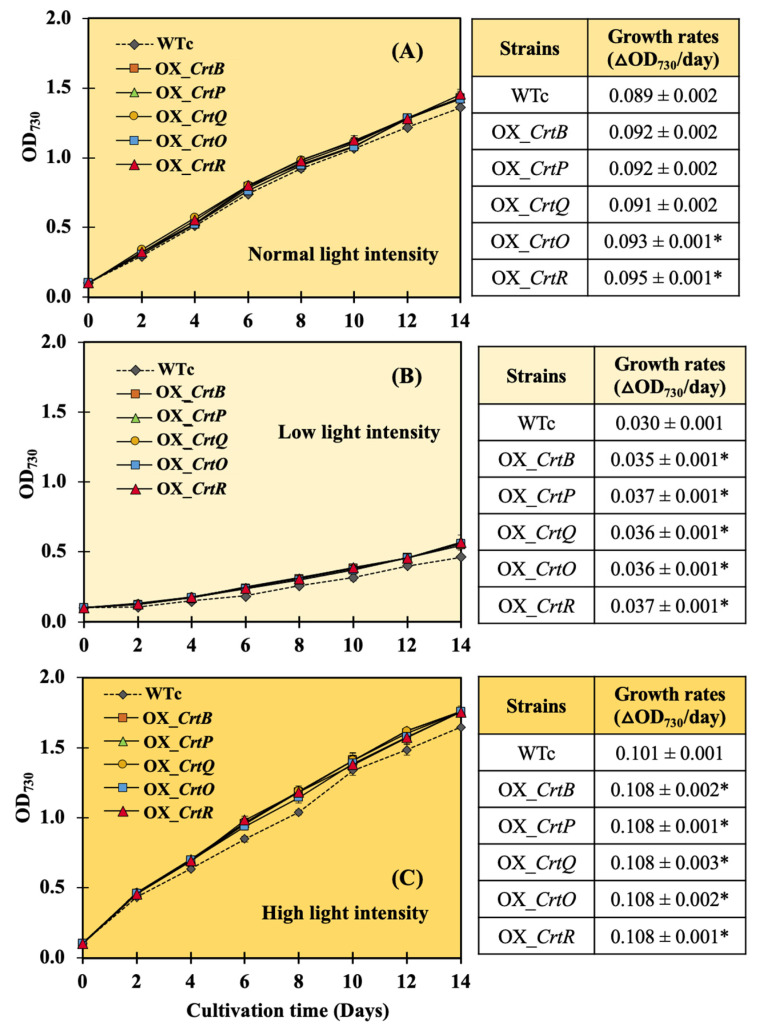
Growth curve and growth rates of *Synechocystis* sp. PCC6803 WTc and engineered strains grown in BG_11_ medium under normal light (**A**), low light (**B**), and high light (**C**) conditions. The error bars represent standard deviations of means (mean ± S.D., n = 3). The statistical difference of the data between the values of WTc and the engineered strain is indicated by an asterisk at * *p* < 0.05.

After exposing all strains to light stresses (Figure 4), low light (LL) conditions apparently affected the growth reduction in all strains, with the lowest growth rates found when comparing those of normal light (NL) and high light (HL) conditions. When cultivated under NL conditions, all OX strains only displayed a higher growth rate than WTc in the late-log phase (Figure 4A). However, they had great levels of growth than WTc under both LL and HL conditions throughout a 14-day treatment period (Figure 4B,C).

**Figure 5:**
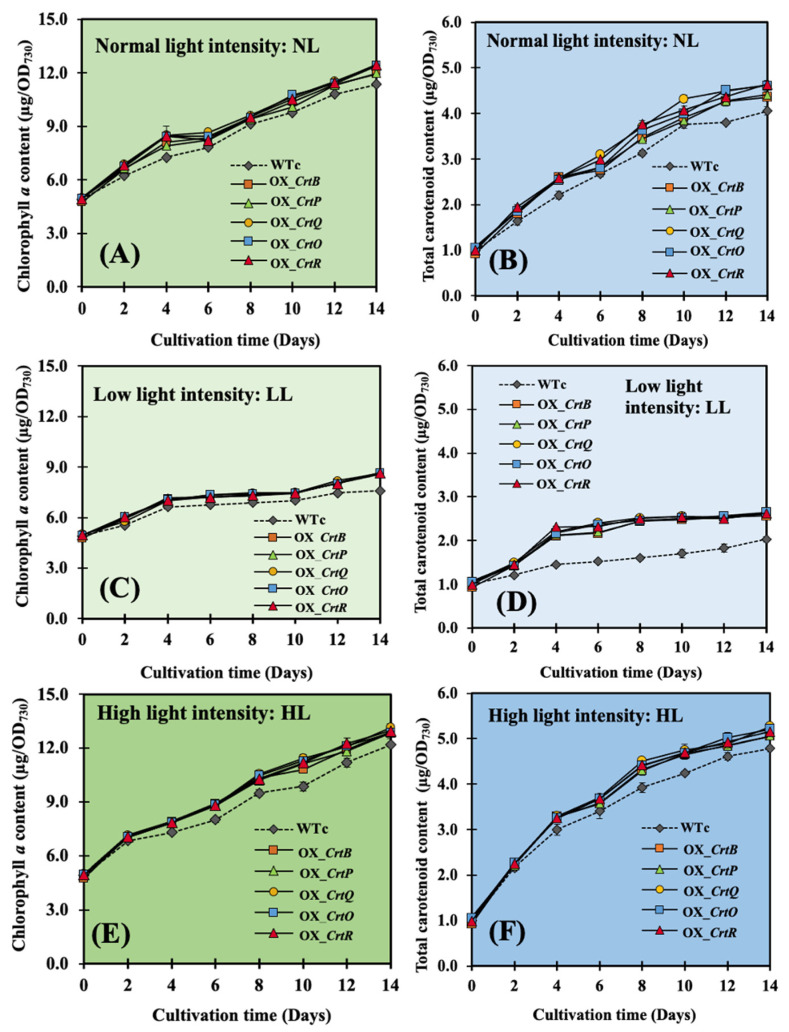
Chlorophyll (Chl) *a* (**A**,**C**,**E**) and total carotenoid (**B**,**D**,**F**) contents of *Synechocystis* sp. PCC6803 WTc and engineered strains. Cells were grown in BG_11_ medium under normal light (**A**,**B**), low light (**C**,**D**), and high light (**E**,**F**) conditions. The error bars represent the standard deviations of means (mean ± S.D., n = 3).

Intriguingly, all OX strains were promoted to accumulate more chlorophyll *a* than WTc (Figure 5). The HL condition resulted in more dark green cell cultures and a higher rise in chlorophyll *a* content than the NL condition after 6 days of treatment (Figure 5A,E). When compared to those under NL conditions, the LL condition undoubtedly lowered the chlorophyll *a* content and the lighter green types of cell culture after both 6 and 12 days of treatment (Figure 5C). On the other hand, all OX strains had a considerable increase in total Car content, as anticipated under all light conditions (Figure 5B,D,F).

**Figure 6:**
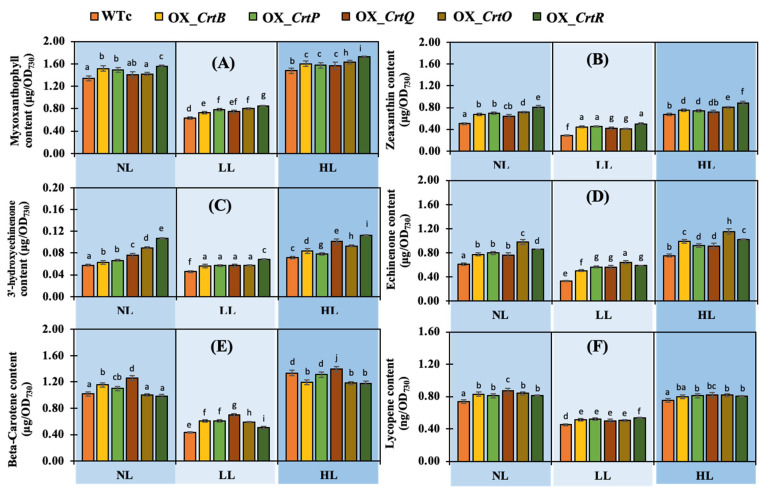
Carotenoid contents, including myxoxanthophyll (**A**), zeaxanthin (**B**), 3′-hydroxy echinenone (**C**), echinenone (**D**), β-carotene (**E**), and lycopene (**F**), of *Synechocystis* sp. PCC6803 WTc and engineered strains. Cells were grown in a BG_11_ medium under normal light (NL), low light (LL), and high light (HL) conditions for 12 days (12 d). The error bars represent the standard deviations of means (mean ± S.D., n = 3). Means with the same letters exhibit nonsignificant differences at a significant level of *p* < 0.05.

Six Car types, including myxoxanthophyll (M), zeaxanthin (Z), 3′-hydroxyechinenone (HE), echinenone (E), β-carotene (bC), and lycopene (L), of all strains were determined under LL and HL conditions (Figure 6). The increased Car accumulation of OX strains was significantly induced by LL and HL light stresses compared with those of WTc (Figure 6A–F). All *Synechocystis* strains apparently contained the highest amount of myxoxanthophyll, followed by β-carotene, echinenone, zeaxanthin, and other minor carotenoids (such as 3′-hydroxyechinenone and lycopene). Although lycopene was accumulated at relatively low levels, all OX strains showed a slight increase as compared to WTc (Figure 6F). This indicates that total Car accumulation was caused by HL stress rather than LL stress. Contrarily, under HL stress, β-carotene levels decreased in OX_*CrtO* and OX_*CrtR*, whereas they remained unaltered under NL conditions (Figure 6E).

**Figure 7:**
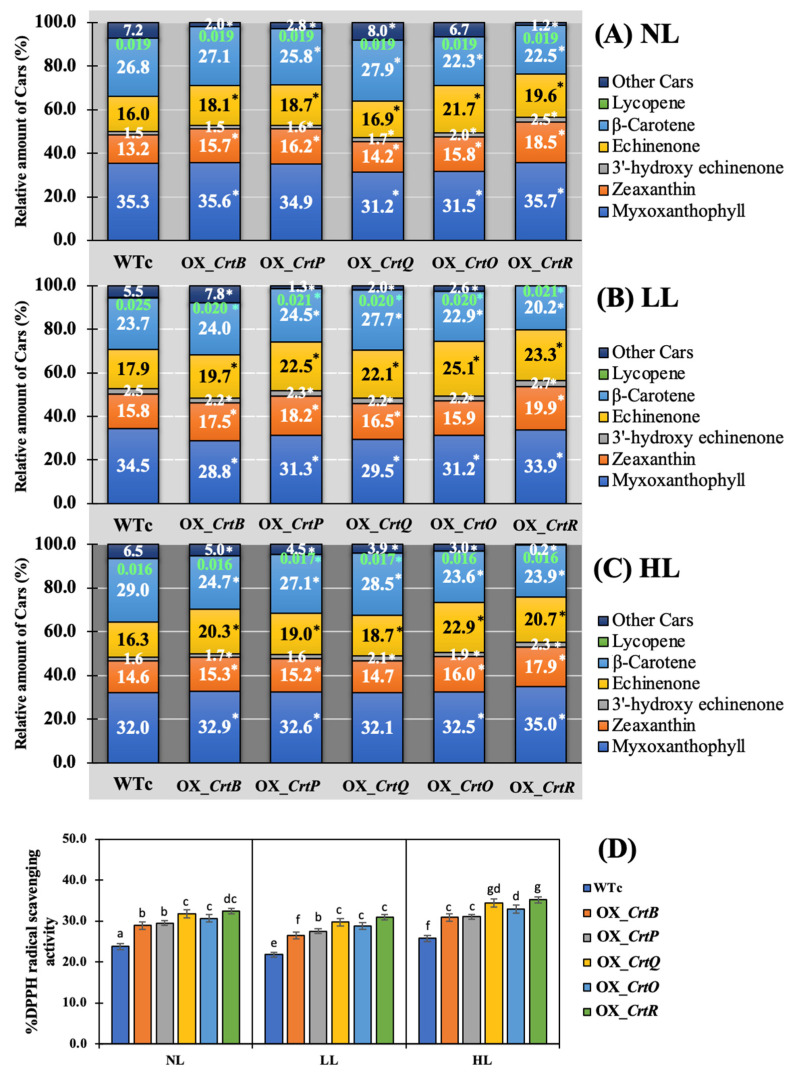
Percentage amounts of carotenoids (**A**–**C**) and %DPPH radical scavenging activities (**D**) of *Synechocystis* sp. PCC6803 WTc and engineered strains. Cells were grown in a BG_11_ medium under (**A**) normal light (NL), (**B**) low light (LL), and (**C**) high light (HL) conditions for 12 days (12 d). The relative amount of Cars was calculated as a percentage relative to total carotenoid content under each light condition. For (**A**), the statistical difference of results between those values of WTc and that engineered strain under each condition is indicated by an asterisk at * *p* < 0.05. For (**D**), the error bars represent the standard deviations of means (mean ± S.D., n = 3). Means with the same letters have nonsignificant differences at a significant level of *p* < 0.05.

In addition, the relative percentage of each Car type was normalized with the total Car content (Figure 7A–C). Although myxoxanthophyll was mostly preserved as a main component in all *Synechocystis* cells, light stress caused cells to balance their Car component by slightly reducing the proportion of myxoxanthophyll. Under NL conditions, all OX strains had higher percentages of zeaxanthin and echinenone, approximately 14–19% and 17–22%, respectively, than WTc, with percentages of about 13 and 16%, respectively (Figure 7A). The HL condition at day 12 of treatment contained a similar pattern of carotenoid composition as those in the NL condition. On the other hand, stronger DPPH radical scavenging activity was shown in all OX strains than WTc under all light conditions (Figure 7D). The highest levels of antioxidant activity were found in the OX_*CrtQ* and OX_*CrtR* strains.

**Figure 8:**
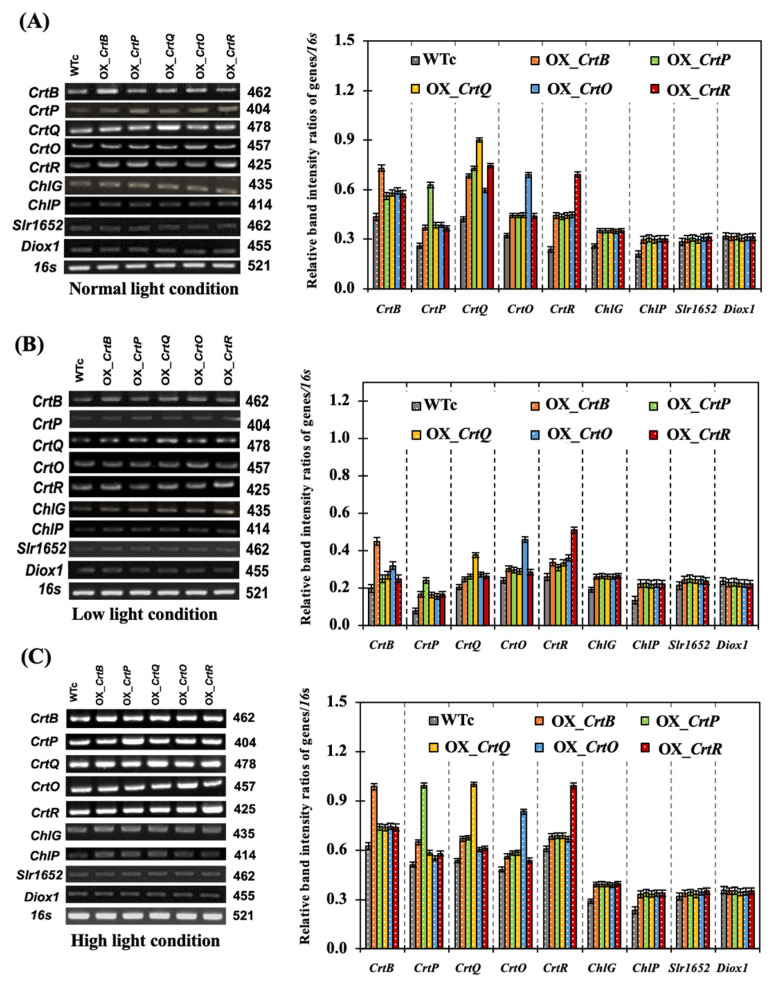
The transcript levels and relative intensity ratios of the *CrtB*, *CrtP*, *CrtQ*, *CrtO*, *CrtR*, *ChlG*, *ChlP*, *Slr1652*, *Diox1*, and *16s* rRNA genes of *Synechocystis* sp. PCC6803 WTc and engineered strains, grown in a BG_11_ medium under normal light (**A**), low light (**B**), and high light (**C**) conditions for 12 days. The error bars represent the standard deviations of means (mean ± S.D., n = 3). All cropped gels were taken from the original images of RT-PCR products on agarose gels, as shown in Supplementary Information Figures S2–S4.

Additionally, the amounts of transcripts from genes involved in the biosynthesis and degradation of Cars and chlorophyll were monitored (Figure 8). The expression of genes involved in Car synthesis was substantially higher on day 12 of treatment under NL and HL conditions than that of genes involved in chlorophyll production and pigment degradation. In all strains cultivated under NL conditions, the transcript levels of the *CrtB* and *CrtQ* genes were the highest of all the genes involved in the Car synthetic pathway (Figure 8A). Furthermore, all five of the genes under investigation, particularly *CrtB* and *CrtR*, exhibited higher mRNA levels as a result of HL stress (Figure 8C). It is curiously interesting that OX strains had higher levels of *ChlG* and *ChlP* transcripts than WTc, in relation to chlorophyll synthesis, under all light conditions (Figure 8). In contrast, there was no difference in the transcript levels of *slr1652*, a gene involved in chlorophyll breakdown, and *Diox1*, a gene involved in Car degradation, under all light conditions.

### 2.3. Enhanced Intracellular Lipid Accumulation and Lung Cancer Cell Treatment

The cooperative function of lipids and Cars, particularly xanthophylls, has recently been found to stabilize the structure of the membrane and the photosystem in cyanobacteria [2,5,28]. In our study, all OX strains with a higher carotenoid content also had higher total lipid contents under all light conditions (Supplementary Information Figure S1). The HL condition at day 12 of treatment significantly increased the total lipid content when compared to the NL and LL conditions on the same day.

**Figure 9:**
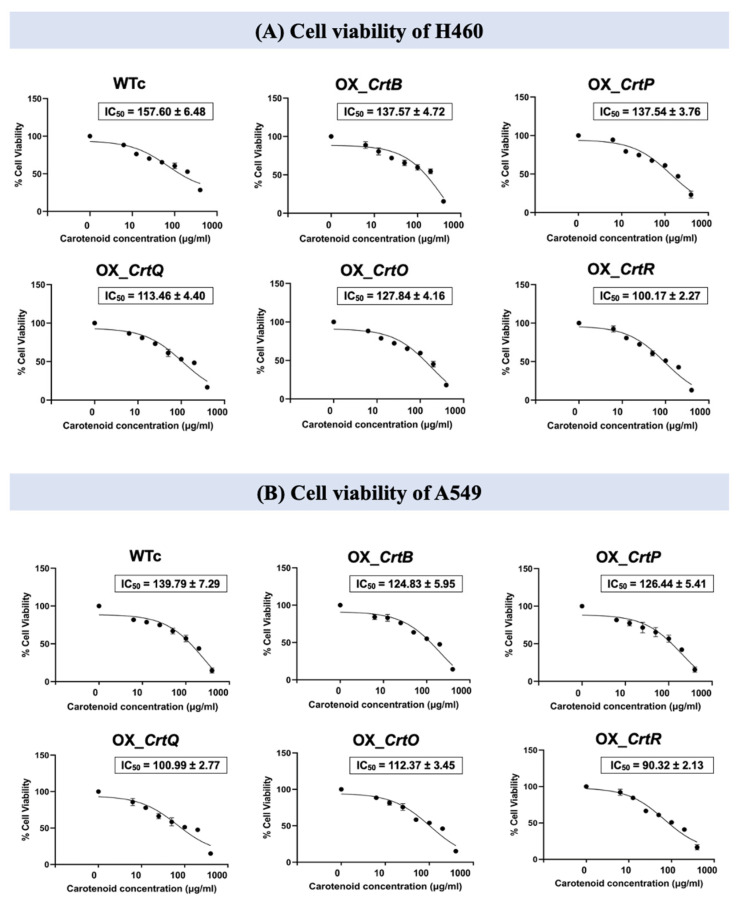
The IC_50_ values of cell viability in lung cancer cells H460 (**A**) and A549 (**B**) after being treated with total carotenoids extracted from *Synechocystis* sp. PCC6803 WTc and engineered strains. Total carotenoids were extracted from cells grown in a BG_11_ medium under high light conditions for 12 days. Lung cancer cells were treated with various concentrations of carotenoid extracts (0–400 µg/mL, circles). The decreasing trendline of %cell viability was shown in black. The error bars represent standard deviations of means (mean ± S.D., n = 3).

**Figure 10:**
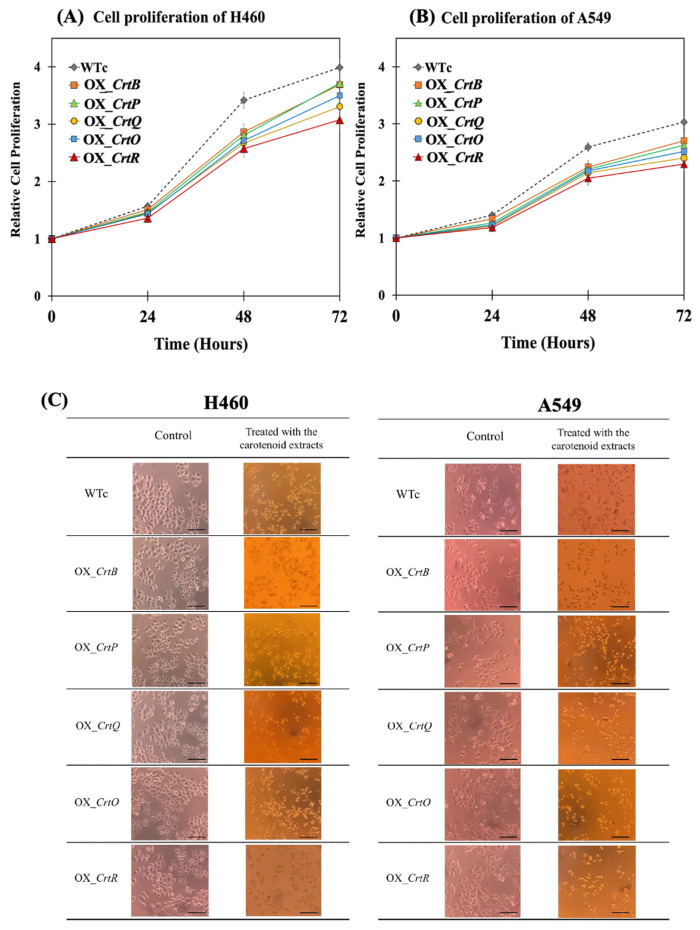
Cell proliferation of lung cancer cells, H460 (**A**) and A549 (**B**), and images of cell lines (**C**) after being treated with total carotenoids extracted from *Synechocystis* sp. PCC6803 WTc and engineered strains. For (**A**,**B**), the cancer cell lines were treated with carotenoid extracts (12.5 µg/mL) for 0, 24, 48, and 72 h at 37 °C. For (**C**), the cancer cell lines were treated with carotenoid extracts (400 µg/mL) for 24 h at 37 °C, and visualized under light microscope with a magnification of 100×, and a scale bar of 50 µm. Total carotenoids were extracted from cells grown in a BG_11_ medium under high light conditions for 12 days. The error bars represent the standard deviations of means (mean ± S.D., n = 3).

Extracted Cars from all strains were evaluated for their cytotoxicity and antiproliferative activities against two lung cancer cell lines (H460 and A549) (Figure 9 and Figure 10). A half-maximal inhibitory concentration (IC_50_) was determined in cancer cell lines (H460 and A549) to evaluate the cytotoxic effect of Car extracts at concentrations of 0–400 µg/mL (Figure 9A,B). The results revealed that the IC_50_ values for Car extracts from OX_*CrtR*, OX_*CrtQ*, OX_*CrtO*, OX_*CrtB*, and OX_*CrtP* in the H460 cell line, with values of 100.17 ± 2.27, 113.46 ± 4.40, 127.84 ± 4.16, 137.57 ± 4.72, and 137.54 ± 3.76 µg/mL, respectively, were lower than those of Car extracts from WTc (157.60 ± 6.48 µg/mL) (Figure 9A). Furthermore, the IC_50_ values for Car extracts from OX_*CrtR*, OX_*CrtQ*, OX_*CrtO*, OX_*CrtP*, and OX_*CrtB* in the A549 cell line were lower than those of Car extracts from WTc (139.79 ± 7.29 µg/mL), with values of 90.32 ± 2.13, 100.99 ± 2.77, 112.37 ± 3.45, 126.44 ± 5.41, and 124.83 ± 5.95 µg/mL, respectively (Figure 9B). Moreover, the relative cell proliferation level of lung cancer cell lines H460 and A549 after being treated with Car extracts for 72 h also decreased when compared with that of WTc (Figure 10A,B). The aberrant cancer cell lines with inhibited growth were discernible under a light microscope resulting from treatment with carotenoid extracts when compared to those of the control group without carotenoids (Figure 10C).

## 3. Discussion

In this study, we found promising results of increased carotenoid (Car) production in the cyanobacterium *Synechocystis* sp. PCC 6803 overexpressing native genes involved in Car biosynthesis with higher antioxidant activity (Figure 1). Five engineered strains, i.e., OX_*CrtB*, OX_*CrtP*, OX_*CrtQ*, OX_*CrtO*, and OX_*CrtR*, significantly promoted Car accumulation and altered Car-type components, particularly zeaxanthin and echinenone, except for OX_*CrtQ*, which also accumulated the increased β-carotene (Figure 3 and Figure 7). All *Synechocystis* cells mostly contained the highest content of myxoxanthophyll due to its vital roles for cell wall structure and thylakoid membrane stabilization [29]. Zeaxanthin can be produced by *Synechocystis* sp. PCC 6803, while other cyanobacteria, such as *Anabaena* sp. PCC 7120 and *Nostoc punctiforme* PCC 73102, are able to generate canthaxanthin and astaxanthin from β-carotene [30,31,32]. We demonstrated that a certain increase in total carotenoid accumulation in all OX strains was found during the late-log phase of cell growth, especially under light stress (Figure 3 and Figure 6). At day 12 of normal cultivation, the native gene overexpression of *slr0088*, encoding CrtO, led to a 1.6-fold increase in echinenone, while an overexpression of *sll1468*, encoding CrtR, contained a 1.6-fold increase in zeaxanthin in comparison with the WT control (Figure 3). In a previous report, a native overexpression of *CrtR* in *Synechocystis* sp. PCC 6803 could induce zeaxanthin with a 2.5-fold increase compared to that in the WT strain [23]. However, we also demonstrated that a higher amount of 3′-hydroxyechinenone, in comparison with WTc, was found in both OX_*CrtO* and OX_*CrtR* strains (Figure 3). In particular, the highest level of 3′-hydroxyechinenone was induced by HL stress in OX_*CrtR*. When cells are exposed to intense light, the photoprotective mechanism at the phycobilisome is induced by the orange carotenoid protein (OCP)-binding hydroxyechinenone [24,33]. The high light intensity stress in this study at 150 µmole photon/m^2^/s was considered a mind photoinhibition inducer, as evidenced by the higher growth and pigment accumulation in Figure 4 and Figure 5. *Synechocystis* cells only experienced severe photoinhibition above 800 µmole photon/m^2^/s, and they displayed an efficient capacity to recover from the complete state of photoinhibition at 1460 µmole photon/m^2^/s when light exposure was reduced to 200 µmole photon/m^2^/s [34]. In addition to the higher accumulation of Cars, we also showed how the types of Cars were distributed differently. In *Synechocystis* wild-type control (Figure 7), the major Car components were myxoxanthophyll (35.3%), β-carotene (26.8%), echinenone (16%), zeaxanthin (13.2%), other minor Cars, such as 3′-hydroxyechinenone (1.5%), lycopene (0.02%), and unidentified carotenoids (7.2%). Mostly, high Cars-producing strains had lowered main components of myxoxanthophyll and β-carotene but increased zeaxanthin, 3-hydroxyechinenone, and echinenone compositions. In addition, after exposure to high light conditions in this study, WT control cells were rearranged for each Car type by inducing proportions of zeaxanthin (from 13.2% to 14.6%), 3′-hydroxyechinenone (from 1.5% to 1.6%), and β-carotene (from 26.8% to 29%), while low light conditions were handled with increased proportions of zeaxanthin (from 13.2% to 15.8%), 3′-hydroxyechinenone (from 1.5% to 2.5%), and echinenone (from 16% to 17.9%) (Figure 7). These findings suggest that zeaxanthin and 3′-hydroxyechinenone are the main Car components that respond to light stress. Zeaxanthin was previously found to enhance high light acclimation by acting as an antioxidant and lipid stabilizer in *Arabidopsis* [35]. High light stress substantially induced the binding of zeaxanthin to specific proteins in photosystems, which enhanced photoprotection and modulated chlorophyll triplet yield [5,36,37]. Both zeaxanthin and echinenone certainly modify photosystem I trimer structure and protect photosystem II repair from singlet oxygen damage in *Synechocystis* sp. PCC6803 [38,39,40]. It was considerably confirmed by our results that OX_*CrtR* with a high zeaxanthin component had the highest capacity for DPPH scavenging activity among all the strains studied (Figure 7D). Additionally, it is worth noting that increased β-carotene composition also helped wild-type cells to cope with high light stress, whereas higher echinenone composition enabled cells to handle low light stress. The native overexpression of *CrtQ* or *slr0940* significantly increased the highest levels of β-carotene and antioxidant activity (Figure 6 and Figure 7). β-carotene accumulation was light-dependently induced, contributing to the acclimation to light stress in *Euglena gracilis* [41]. On the other hand, the transcript results indicated that light stress had a close relationship with enhanced carotenoid synthesis rather than degradation and chlorophyll metabolism, particularly under high light conditions (Figure 8).

We also demonstrated that carotenoid synthetic gene overexpression could improve intracellular lipid contents (Supplementary Information Figure S1). An increase in the light intensity during cultivation resulted in an increase in lipids and fatty acid production, regulating membrane integrity [42,43,44]. The cooperative function of lipids and carotenoids led cells to acclimate their membrane structure and function against light stress [1,28,45,46]. On the other hand, the carotenoid extracts from all engineered strains were applied to treat lung cancer cell lines H460 and A549. Lung cancer has been the first and foremost leading cause of death around the world. A higher intake of carotenoids could lower the risk of lung cancer [17,47,48]. Our findings demonstrated that the carotenoid extracts from all engineered *Synechocystis* sp. PCC 6803 strains significantly decreased the viability and proliferation of lung cancer cell lines, particularly OX_*CrtR* and OX_*CrtQ* (Figure 9 and Figure 10). *Synechocystis* sp. PCC 6803 overexpressing *CrtR* with high antioxidant activity showed the lowest level of IC_50_ at 100.17 and 90.32 µg/mL on H460 and A549 cell lines, respectively. The anti-cancer therapeutic targets of Cars include cell cycle arrest, an apoptosis-inducing effect, an anti-metastatic effect, and an anti-angiogenic effect in cancer cells [17], as well as modulating oxidative stress and redox balance [16]. For all Car types, they have synergistic functions of antioxidant activity to modulate ROS levels and have also shown pro-oxidant effects in cancer cells [16,49]. β-carotene and lycopene were also considered potential anti-cancer compounds previously found to inhibit tumor cell growth by interfering at different phases of the cell cycle and generate apoptosis-inducing effects in cancer cells, such as the G1 phase arrest of human promyelocytic leukemia (HL-60) cells and the G0/G1 phase arrest of HL-60 cells, respectively [50,51]. In addition, β-carotene played an effective anti-metastatic role in lung cancer cells B16F-10 by reducing collagen, hydoxylproline, uronic acid, and hexamine contents [52]. On the other hand, zeaxanthin treated uveal melanoma cell lines by inducing apoptosis by activating the intrinsic apoptosis signaling pathway [53]. In accordance with our findings, *Synechocystis* sp. PCC6803 strains with *CrtR* and *CrtQ* overexpression, respectively, had the highest amounts of zeaxanthin and β-carotene. These two OX strains exhibited a certain increase in antioxidant activity, and their carotenoid extracts had the highest capacity to treat lung cancer cells in vitro.

## 4. Materials and Methods

### 4.1. Strains and Culture Conditions

Cyanobacterium *Synechocystis* sp. PCC 6803 wild-type (WT) strains and all engineered strains (Table 1) were grown in the BG_11_ medium. The growth condition was performed at 27–30 °C under continuous white light illumination with various light intensities, including low light (LL; 10 µmole photon/m^2^/s), normal light (NL; 50 µmole photon/m^2^/s) and high light (HL; 150 µmole photon/m^2^/s) conditions. The cell culture flask with an initial optical density (OD) of about 0.1 at 730 nm was placed on a rotary shaker (160 rpm) for 14 days. Cell growth was spectrophotometrically measured at OD_730_. The host propagation, *Escherichia coli* DH5α strain, was grown either on a Luria–Bertani (LB) agar plate or its liquid medium at 37 °C.

### 4.2. Constructions of Recombinant Plasmids

The recombinant plasmids (Table 1), including pEERM_*CrtB*, pEERM_*CrtP*, pEERM_*CrtQ*, pEERM_*CrtO*, and pEERM_*CrtR*, were constructed to generate OX_*CrtB*, OX_*CrtP*, OX_*CrtQ*, OX_*CrtO*, and OX_*CrtR* strains, respectively. Initially, five target gene fragments involved in the carotenoid biosynthetic pathway were amplified by PCR using each specific pair of primers, as listed in Supplementary Information Table S1, and genomic DNA as a template. Each gene fragment was then ligated into the pEERM vector with the *psbA2* promoter, which could practically let the engineered cells grow under high light conditions [54], generating the expected five recombinant plasmids listed in Table 1. pEERM_*CrtB* was generated via the ligation of the *CrtB* gene fragment amplified by FW_*CrtB* and RV_*CrtB* (Supplementary Information Table S1) with the pEERM vector in the *PstI* restriction site. For pEERM_*CrtP* and pEERM_*CrtQ*, the *CrtP* and *CrtQ* gene fragments, amplified by a pair of FW_*CrtP* and RV_*CrtP* primers and another pair of FW_*CrtQ* and RV_*CrtQ* (Supplementary Information Table S1), respectively, were separately introduced in between *XbaI* and *SpeI* restriction sites in the pEERM vector. Moreover, pEERM_*CrtO* and pEERM_*CrtR* were generated by ligating *CrtO* and *CrtE* gene fragments, amplified by PCR using a pair of FW_*CrtO* and RV_*CrtO* and another pair of FW_*CrtR* and RV_*CrtR* primers, respectively (Supplementary Information Table S1), in between *SpeI* and *PstI* sites in the pEERM vector.

### 4.3. Natural Transformation of Synechocystis sp. PCC 6803 Cells

The recombinant plasmids (Table 1) were transformed into *Synechocystis* sp. PCC 6803 WT cells by natural transformation, thereby generating OX_*CrtB*, OX_*CrtP*, OX_*CrtQ*, OX_*CrtO*, and OX_*CrtR*. To construct *Synechocystis* WT control (WTc) or *Synechocystis* WT containing the *Cm^R^* cassettle gene, the empty pEERM vector was transformed into *Synechocystis* WT cells. First, the host cell suspension was prepared. Then, 50 mL of *Synechocystis* sp. PCC 6803 WT cell culture with an OD_730_ of about 0.5 was harvested via centrifugation at 5500 rpm (2870× *g*) for 15 min. Cell pellets were resuspended in a fresh BG_11_ medium (500 µL). After that, at least 10 µL of the recombinant plasmids were mixed with the *Synechocystis* host cell suspension and incubated in the culture room overnight. Then, the sample mixture was spread on a BG_11_ agar plate containing 10 µg/mL of chloramphenicol. After several weeks, the colonies that survived on antibiotic plates were picked and streaked on a new BG_11_ agar plate containing 20, and later 30 µg/mL, of chloramphenicol. The obtained transformants were confirmed for gene size, location, and segregation by PCR using many specific pairs of primers, as shown in the Supplementary Information Table S1.

### 4.4. Determinations of Chlorophyll a and Total Carotenoid Contents

The chlorophyll *a* and total carotenoid contents were analyzed using the modified method from [55]. Next, 1 mL of cell culture was extracted using the acetone:water mixture (4:1) as a solvent and incubated for 2 min. After centrifugation at 5500 rpm (2870× *g*) for 5 min, the supernatant was spectrophotometrically measured at wavelengths of 470.0, 646.6, and 663.6 nm, respectively. Contents of chlorophyll *a* and carotenoids were calculated using the following equations:

Chlorophyll *a* content (µg/OD_730_) = [(12.25 × A_663.6_) − (2.25 × A_646.6_)]/OD_730_

Chlorophyll *b* content (µg/OD_730_) = [(20.31 × A_646.6_) − (4.91 × A_663.6_)]/OD_730_

Total carotenoid content (µg/OD_730_) = [1000A_470_ − (2.27 × Chlorophyll *a* content) − (81.4 × Chlorophyll *b* content)]/227 × OD_730_

### 4.5. Quantitative Analysis of Carotenoids by HPLC

Then, 50 mL of *Synechocystis* cell culture was harvested by centrifugation at 5500 rpm (2870× *g*) for 10 min. Before starting solvent extraction, the cell suspension of all samples was diluted to an OD_730_ of about 0.5. Then, cell pellets from 3 mL of diluted cell suspension were collected after centrifugation again. The intracellular pigments were then extracted by absolute methanol (1 mL), and centrifuged at 12,000 rpm (21,009× *g*) and 4 °C for 5 min. After that, the supernatant was collected for further analysis. Samples of extracted intracellular pigments were detected using high-performance liquid chromatography (HPLC) (Shimadzu HPLC LGE System, Kyoto, Japan) using a C18 column, 150 × 46 mm (GL-Sciences, Tokyo, Japan). The HPLC condition was set at 15 °C, with a flow rate of 1 mL/min using the solvent system described by [1]. This solvent system consisted of an isocratic elution with a mixture of solvent A, acetonitrile: methanol: Tris (0.1 M, pH 8.0) (89/9.5/1.5, *v*/*v*), for 4 min; a linear gradient from solvent A to solvent B, methanol: hexane (4:1, *v*/*v*), for 2.5 min; an isocratic elution with solvent B for 11.5 min; a linear gradient of solvent B to solvent A for 1 min; and a final isocratic elution with solvent A for 9 min. The separated pigments were detected at 440 nm using a UV/VIS detector. The carotenoid contents, including myxoxanthophyll, zeaxanthin, 3′-hydroxy echinenone, echinenone, and β-carotene, were calculated using the following equations from [23]: C_car_ = C_chl_ × [(ε_chl_ × A_car_)/(ε_car_ × A_chl_)], where C_chl_ = the chlorophyll concentration in the pigments extracts was determined by the extinction coefficient of chlorophyll *a* using 820, and the absorbance of the pigment extract at 663 nm. ε_chl_ and ε_car_ = the specific extinction coefficients of the chlorophyll *a* and the carotenoids at 440 nm, respectively, were taken from [56,57]. A_chl_ and A_car_ = the chlorophyll *a* and the carotenoid peak areas on the chromatogram, respectively, were detected at 440 nm. The relative amount of each carotenoid type was calculated as a percentage to the total carotenoid content under each light condition.

### 4.6. Determination of Lycopene Content

Lycopene content was determined using the modified method from [58]. Then, 1 mL of cell culture was extracted using the acetone:hexane solvent mixture (2:3) and incubated at room temperature for 2 min. After that, the supernatant, obtained after centrifugation at 5500 rpm (2870× *g*) for 10 min, was spectrophotometrically measured at four wavelengths of 453, 505, 645, and 663 nm, respectively. Lycopene content was calculated using the following equation: Lycopene content (µg/OD_730_) = [(−0.0485 × A_663_) + (0.204 × A_645_) (0.372 × A_505_) − (0.0806 × A_453_)]/100,000 × OD_730_, where A = absorbance.

### 4.7. Total RNA Extraction and Reverse Transcription–Polymerase Chain Reaction (RT-PCR)

The total RNAs were extracted from all strains using the TRIzol^®^ reagent (Invitrogen, Life Technologies Corporation, Carlsbad, CA, USA). The purified RNAs (1 µg) were converted to cDNA using the SuperScript III First-Strand Synthesis Kit (Invitrogen, Carlsbad, CA, USA). Then, the obtained cDNA was used as a template for PCR with various pairs of specific primers, as listed in Supplementary Information Table S1. We monitored the *CrtB*, *CrtP*, *CrtQ*, *CrtR*, and *CrtO* genes in relation to the carotenoid biosynthetic pathway; the *Diox1* gene in carotenoid degradation or retinal biosynthesis; the *ChlP* and *ChlG* genes in chlorophyll biosynthesis; and the *Slr1652* gene in chlorophyll degradation. The *16s* rRNA was used as a reference. The PCR reaction mixture using KOD OneTM Master Mix (Toyobo, Osaka, Japan) and its conditions were performed by suitable cycles of each gene at 98 °C for 10 s, at both a primer melting temperature (Tm, Supplementary Information Table S1) for 5 s and 68 °C for 5 s. Then, the PCR products were checked by 1% (*w*/*v*) agarose gel electrophoresis. The quantification of band intensity was detected by Syngene^®^ Gel Documentation (Syngene, Frederick, MD, USA).

### 4.8. Determination of DPPH Radical Scavenging Activity

The free radical scavenging activity of pigment extracts from all strains was measured using a DPPH assay (modified from [59]). Then, 1 mL of cell culture was harvested by centrifugation at 5500 rpm (2870× *g*) for 10 min. Pigment extracts were obtained from cell pellets after they were incubated with a solvent mixture of acetone and water (4:1) at room temperature for 10 min. The 2,2-diphenyl-1-picrylhydrazyl (DPPH) concentration was prepared as 0.1 mM in ethanol. The DPPH solution (500 µL) was mixed with the cell-free pigment extract (500 µL). The reaction mixture was incubated at room temperature for 30 min. After that, the absorbance at 517 nm was spectrophotometrically measured. An ascorbic acid was used as a standard. The antioxidant activity was calculated using the equation; DPPH radical scavenging activity (%) = A_0_ − A_1_/A_0_ × 100, where A_0_ is the absorbance of the control reaction and A_1_ is the absorbance of the sample.

### 4.9. Cell Line Culture

NSCLC cell lines A549 (CCL-185) and H460 (HTB-177) were obtained from the American Type Culture Collection (ATCC; Manassas, VA, USA). The H460 cell line was obtained from the pleural fluid of a male patient with large cell lung cancer, while the A549 cell line was obtained from an explant culture of lung carcinomatous tissue from an old male patient. The A549 cells were cultured in Dulbecco’s modified Eagle medium (DMEM), while the H460 cells were cultured in Roswell Park Memorial Institute 1640 medium (RPMI-1640). Both media were supplemented with 10% fetal bovine serum albumin, 2 mM L-glutamine, and 100 U/mL of penicillin–streptomycin (Invitrogen, Carlsbad, CA, USA). Cell culture was maintained in a humidified atmosphere containing 5% CO_2_ at 37 °C. The culture medium and trypsinization were renewed every 2–3 days. 3-(4,5-dimethylthiazol-2-yl)-2,5-diphenyltetrazolium bromide (MTT) (Invitrogen, Carlsbad, CA, USA) solution (5 mg/mL) was freshly prepared in phosphate-buffered saline (PBS).

### 4.10. Cytotoxicity and Cell Proliferative Assay

Total carotenoids were extracted from all strains by a solvent mixture of hexane:acetone:ehanol (50:25:25, *v*/*v*/*v*) and a subsequent 1 M KOH solution to remove chlorophyll (modified from [60]). After the centrifugation was performed at 5500 rpm (2870× *g*), 4 °C for 10 min, the upper fraction layer of hexane containing total carotenoid extracts was carefully collected, and the solvent was evaporated under nitrogen gas at room temperature. Extracted carotenoid powder was dissolved in a PBS buffer with 0.25% (*v*/*v*) dimethyl sulfoxide (DMSO; Sigma, St. Louis, MO, USA), the maximum DMSO concentration that did not interfere with the assays in various concentrations (0–400 µg/mL).

The cytotoxicity of the extracts was determined by the MTT assay (modified from [14,61]). Cell lines were seeded into 96-well plates at a density of 10,000 cells per well and incubated at 37 °C overnight for cell attachment purposes. The cells were then treated with various concentrations of carotenoid extracts ranging from 0 to 400 µg/mL for 24 h. After the incubation period of 24 h, 10 µL of MTT solution (5 mg/mL) was added to each well and incubated at 37 °C for 4 h. After the incubation period, the resulting formazan crystals were formed, and dissolved in 100 µL of DMSO. After that, the colored product was spectrophotometrically measured at 570 nm. The results from the MTT assay represented the IC_50_ value of the different carotenoid extracts from all strains by analyzing their effect on cell viability.

Cell proliferative assay was determined by the MTT assay (modified from [62]). Cell lines were seeded at a density of 3000 cells per well into 96-well plates. Cells were treated with non-toxic concentrations of the carotenoid extracts (12.5 µg/mL) and incubated at 37 °C for 24, 48, and 72 h. After the incubation at each elapsed time, 10 µL of MTT (5 mg/mL) was added into each well and incubated at 37 °C for 4 h. The formazan crystals were formed and later dissolved by 100 µL of DMSO. After that, the sample was spectrophotometrically measured at 570 nm. Relative cell proliferation was calculated by dividing the OD_570_ of the treated group by the OD_570_ of the control.

### 4.11. Statistical Analysis

Statistical analyses were performed using SPSS version 29.0, licensed by Chulalongkorn University. The differences in biochemical parameters and molecular responses among the treatments were analyzed using one-way ANOVA, followed by Tukey’s HSD test for the multiple comparison tests. The *t*-test was performed for an independent two-pair comparison test. Non-parametric tests were used for the data which did meet the assumption of normality. The significant differences were accepted at *p* < 0.05.

## 5. Conclusions

Five engineered *Synechocystis* sp. PCC 6803 strains with high carotenoid accumulation and antioxidant activity were achieved by overexpressing five carotenoid biosynthetic genes, thereby generating OX_*CrtB*, OX_*CrtP*, OX_*CrtQ*, OX_*CrtO*, and OX_*CrtR*. The increased levels of zeaxanthin and echinenone were mostly found in all engineered strains, whereas myxoxanthophyll was maintained as the main carotenoid type. An increase in carotenoid content was especially induced under high light stress at 150 µmole photon/m^2^/s, except for β-carotene. Changes in the components of all carotenoid types were considered a marker that responded to light stress. Additionally, all modified strains were more susceptible to high light-stress-induced lipid accumulation. It is worth noting that the extracted carotenoids with high antioxidant activity from all engineered strains efficiently treated lung cancer cells H460 and A549 by decreasing their cell viability and proliferation, particularly OX_*CrtR* and OX_*CrtQ*, with high levels of zeaxanthin and β-carotene, respectively. Although the mechanisms of all carotenoid types treating cancer cells are still not completely elucidated, this study highlights a promising step towards the cyanobacterial application on health and cancer treatment aspects from renewable biosources. It would be useful to characterize and gain more understanding about carotenoid mechanisms or perform animal model testing on the carotenoid extracts in vivo.

## Figures and Tables

**Table 1:** Strains and plasmids used in this study.

Name	Relevant Genotype	Reference
Cyanobacterial strains		
*Synechocystis* sp. PCC 6803	Wild type	Pasteur Culture Collection
Wild-type control (WTc)	*Cm^R^* integrated at the region of a native *psbA2* gene in the *Synechocystis* genome	This study
OX_*CrtB*	*CrtB* and *Cm^R^* integrated at the region of a native *psbA2* gene in the *Synechocystis* genome	This study
OX_*CrtP*	*CrtP* and *Cm^R^* integrated at the region of a native *psbA2* gene in the *Synechocystis* genome	This study
OX_*CrtQ*	*CrtQ* and *Cm^R^* integrated at the region of a native *psbA2* gene in the *Synechocystis* genome	This study
OX_*CrtO*	*CrtO* and *Cm^R^* integrated at the region of a native *psbA2* gene in the *Synechocystis* genome	This study
OX_*CrtR*	*CrtR* and *Cm^R^* integrated at the region of a native *psbA2* gene in the *Synechocystis* genome	This study
Plasmids		
pEERM	P_psbA2_-*Cm^R^*; plasmid containing the flaking region of a *psbA2* gene	This study
pEERM_*CrtB*	P_psbA2_-*CrtB*; integrated between the *PstI* sites of pEERM	This study
pEERM_*CrtP*	P_psbA2_-*CrtP*; integrated between the *XbaI* and *SpeI* sites of pEERM	This study
pEERM_*CrtQ*	P_psbA2_-*CrtQ*; integrated between the *XbaI* and *SpeI* sites of pEERM	This study
pEERM_*CrtO*	P_psbA2_-*CrtO*; integrated between the *SpeI* and *PstI* sites of pEERM	This study
pEERM_*CrtR*	P_psbA2_-*CrtR*; integrated between the *SpeI* and *PstI* sites of pEERM	This study

P_psbA2_, strong *psbA2* promoter; *Cm^R^*, chloramphenicol resistance cassette.

## Data Availability

Not applicable.

## Supplementary Materials

The supporting information can be downloaded at: https://www.mdpi.com/article/10.3390/ijms24119370/s1.

## Abbreviations

Cars	carotenoids
Cm	chloramphenicol
DMEM	Dulbecco’s modified Eagle medium
DMSO	dimethyl sulfoxide
DPPH	2,2-diphenyl-1-picrylhydrazyl
h	hour
kb	kilobase
LB medium	Luria–Bertani medium
m	meter
M	molar
mM	millimolar
mg	milligram
mL	milliliter
min	minutes
µg	microgram
µL	microliter
nm	nanometer
MTT	3-(4,5-dimethylthiazol-2-yl)-2,5-diphenyltetrazolium bromide
OCP	orange carotenoid protein
OD	optical density
OX strain	overexpressed strain
PCR	polymerase chain reaction
ROS	reactive oxygen species
Rpm	revolutions per minute
RPMI-1640	Roswell Park Memorial Institute 1640
s	second
WT	wild type
