# Supplementary material for: Overexpressing Carotenoid Biosynthetic Genes in Synechocystis sp. PCC 6803 Improved Intracellular Pigments and Antioxidant Activity, Which Can Decrease the Viability and Proliferation of Lung Cancer Cells In Vitro
Source: Int J Mol Sci. 2023 May 27;24(11):9370. doi: 10.3390/ijms24119370 (PMC10253290; doi:10.3390/ijms24119370)
Supplement: Supplementary file 1 [file ijms-24-09370-s001.zip › ijms-2406982-supplementary.pdf]

Supplementary Information, Table S1 Primers used in this study

Name	Sequence (5' to 3')	Purpose of primer	PCR cycles/ T_m	Expected size (bp)
FW_*CrtB*	TAGAGACTGCAGTGCCCAACTTTTACC	PCR for *CrtB*		1330
RV_*CrtB*	TAGAGACTGCAGAGTGAGTCCTTAGC	PCR for *CrtB*		
FW_*CrtP*	TAGAGATCTAGAGGCTATCTTGGCAAAGT	PCR for *CrtP*		1619
RV_*CrtP*	TAGAGAACTAGTTGGTAACTGCACGCTG	PCR for *CrtP*		
FW_*CrtQ*	TAGAGATCTAGAACAACCTTGGCGCTT	PCR for *CrtP*		1670
RV_*CrtQ*	TAGAGAACTAGTCAGACCACAGTTGCCA	PCR for *CrtP*		
FW_*CrtO*	TAGAGAACTAGTGCACGATCCTCATCAA	PCR for *CrtP*		1829
RV_*CrtO*	TAGAGACTGCAGCCTAACATGGTAAAC	PCR for *CrtP*		
FW_*CrtR*	TAGAGAACTAGTCCCTGGTCTCCTACAT	PCR for *CrtP*		1139
RV_*CrtR*	TAGAGACTGCAGGGTCTGGGGAAT	PCR for *CrtP*		
Up*psbA2*	TGCCTGTCAGCAAAACAACTT	Colony PCR		640
DS*psbA2*	CGAGGGCAATCATCAATTCCG	Colony PCR		640
FW_Cm^R	GAGTTGATCGGCACGTAAG	Colony PCR		899
RV_Cm^R	CTCGAGGCTTGGATTCTCTCAC	Colony PCR		
RT_FW_16S	AGTTCTGACGGTACCTGATGA	RT-PCR for 16S	16 cycles/ 56 ºC	521
RT_RV_16S	GTCAAGCCTTGGTAAGGTTCT	RT_PCR for 16S		
RT_FW_*CrtB*	TGACCAATATTCTGCGGGACGTT	RT_PCR for *CrtB*	25 cycles/ 60ºC	462
RT_RV_*CrtB*	TAGAGACTGCAGAGTGAGTCCTTAGC	RT_PCR for *CrtB*		
RT_FW_*CrtP*	ATCAAGCAACTCTTTCCCCAACACT	RT_PCR for *CrtP*	25 cycles/ 60ºC	404
RT_RV_*CrtP*	TAGAGAACTAGTTGGTAACTGCACGCTG	RT_PCR for *CrtP*		
RT_FW_*CrtQ*	CCCAGGGGAAGGTTCCCTATTACAACT	RT_PCR for *CrtQ*	24 cycles/ 58ºC	478
RT_RV_*CrtQ*	TAGAGAACTAGTCAGACCACAGTTGCCA	RT_PCR for *CrtQ*		
RT_FW_*CrtO*	GGGCACAGGTTGGACCGATGAGTT	RT_PCR for *CrtO*	27 cycles/ 58ºC	457
RT_RV_*CrtO*	TAGAGACTGCAGCCTAACATGGTAAAC	RT_PCR for *CrtO*		
RT_FW_*CrtR*	AGCCCAACATTAAGGCACTACCGT	RT_PCR for *CrtR*	25 cycles/ 56ºC	425
RT_RV_*CrtR*	TAGAGACTGCAGGGTCTGGGGAAT	RT_PCR for *CrtR*		
RT_FW_*ChlP*	TGGTTGGTCGAGTAGCCCTAG	RT_PCR for *ChlP*	25 cycles/ 58ºC	414
RT_RV_*ChlP*	TTAAGGGGCTAAAGCGTTACCC	RT_PCR for *ChlP*		
RT_FW_*ChlG*	TACATTGCCCTACCGTGGTGG	RT_PCR for *ChlG*	27 cycles/ 58ºC	435
RT_RV_*ChlG*	TCAAATCCCCGCATGGCCTA	RT_PCR for *ChlG*		
RT_FW_*Slr1652*	GCAGTGCTCTCCTATCGTTTG	RT_PCR for *Slr1652*	29 cycles/ 55ºC	462
RT_RV_*Slr1652*	CTATCCCAACCAAAGATAGCTACC	RT_PCR for *Slr1652*		
RT_FW_*Diox1*	AATTTAGATCCCGGACAGTTATGG	RT_PCR for *Diox1*	27 cycles/ 56ºC	455
RT_RV_*Diox1*	TCAAGTCTGGGCCCAGGAAC	RT_PCR for *Diox1*		

Supplementary Information, Figure S1

Intracellular lipid contents of *Synechocystis* sp. PCC6803 WTc and engineered strains, grown in BG_{11} medium under normal light (**A**), low light (**B**), and high light (**C**) conditions for 0 (the start of experiment), 6 and 12 days. The error bars represent standard deviations of means (mean ± S.D., n=3). Means with the same letters have nonsignificant differences at a significant level of $P < 0.05$.

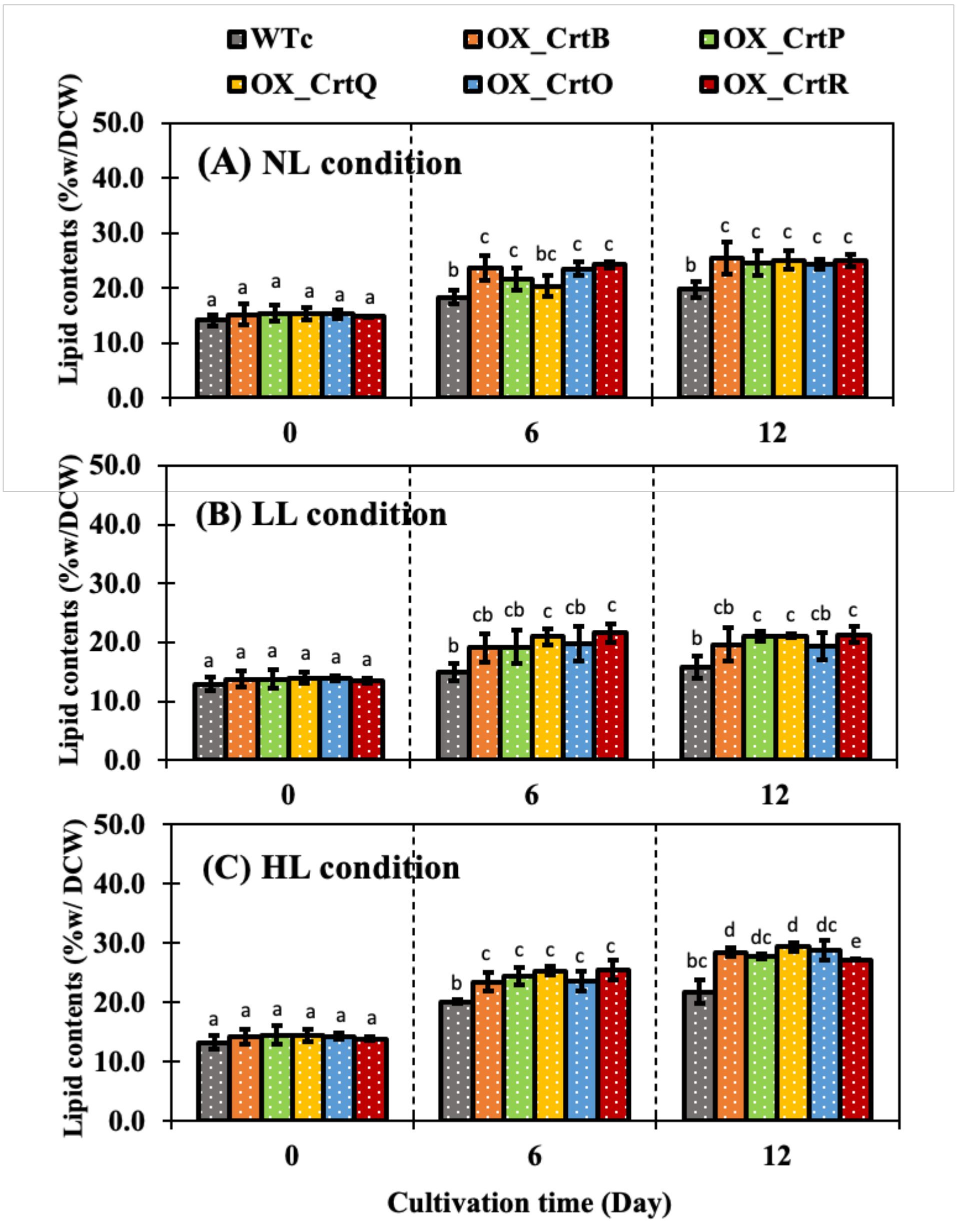

Supplementary Information, Figure S2

Agarose gel electrophoresis of RT-PCR product of carotenoids biosynthetic genes and related gene transcripts in all of *Synechocystis* sp. PCC 6803 strains grown under normal light condition. The *16s* rRNA transcript was used as the reference.

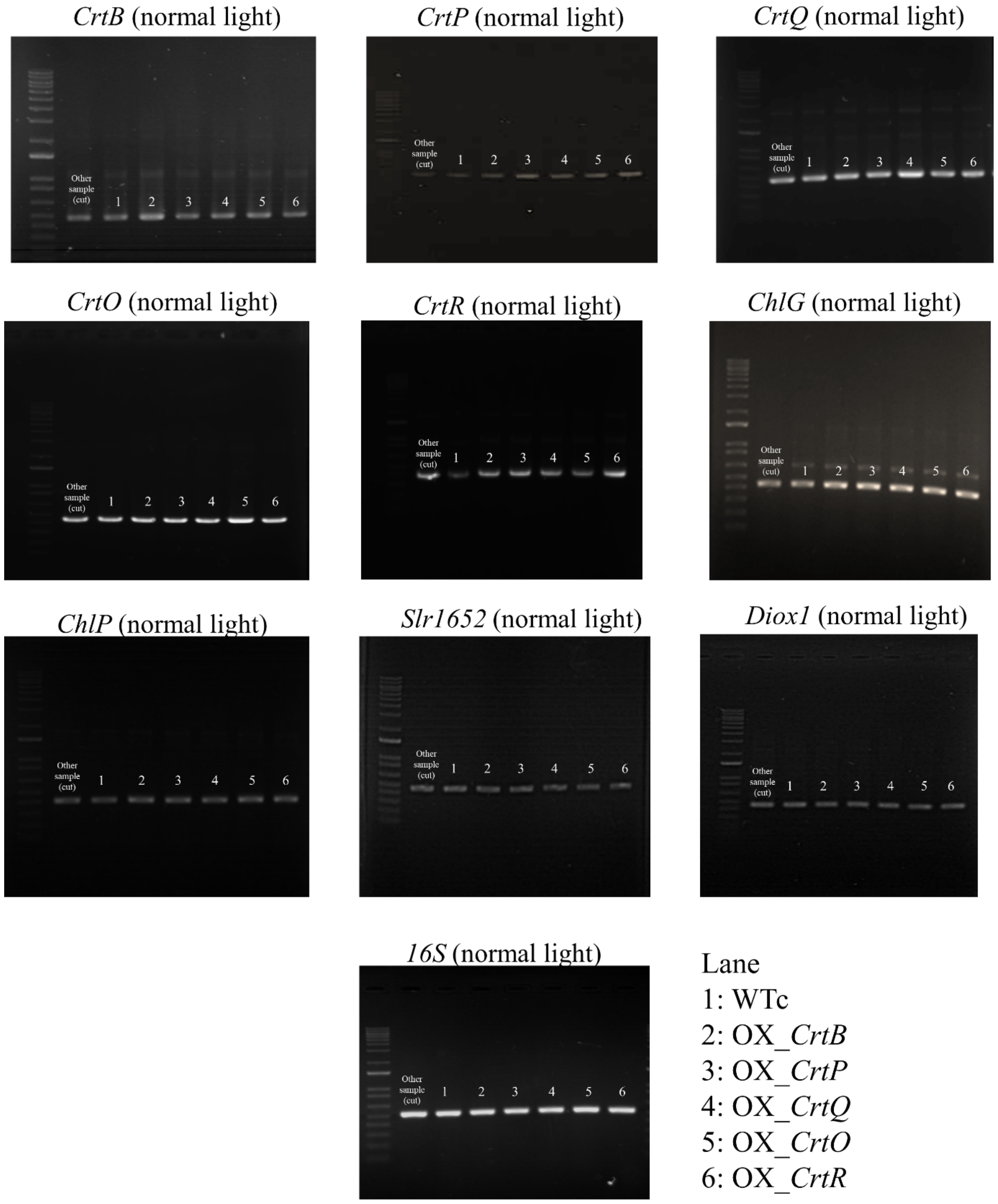

Supplementary Information, Figure S3
Agarose gel electrophoresis of RT-PCR product of carotenoids biosynthetic genes and related gene transcripts in all of *Synechocystis* sp. PCC 6803 strains grown under low light condition. The *16s* rRNA transcript was used as the reference.

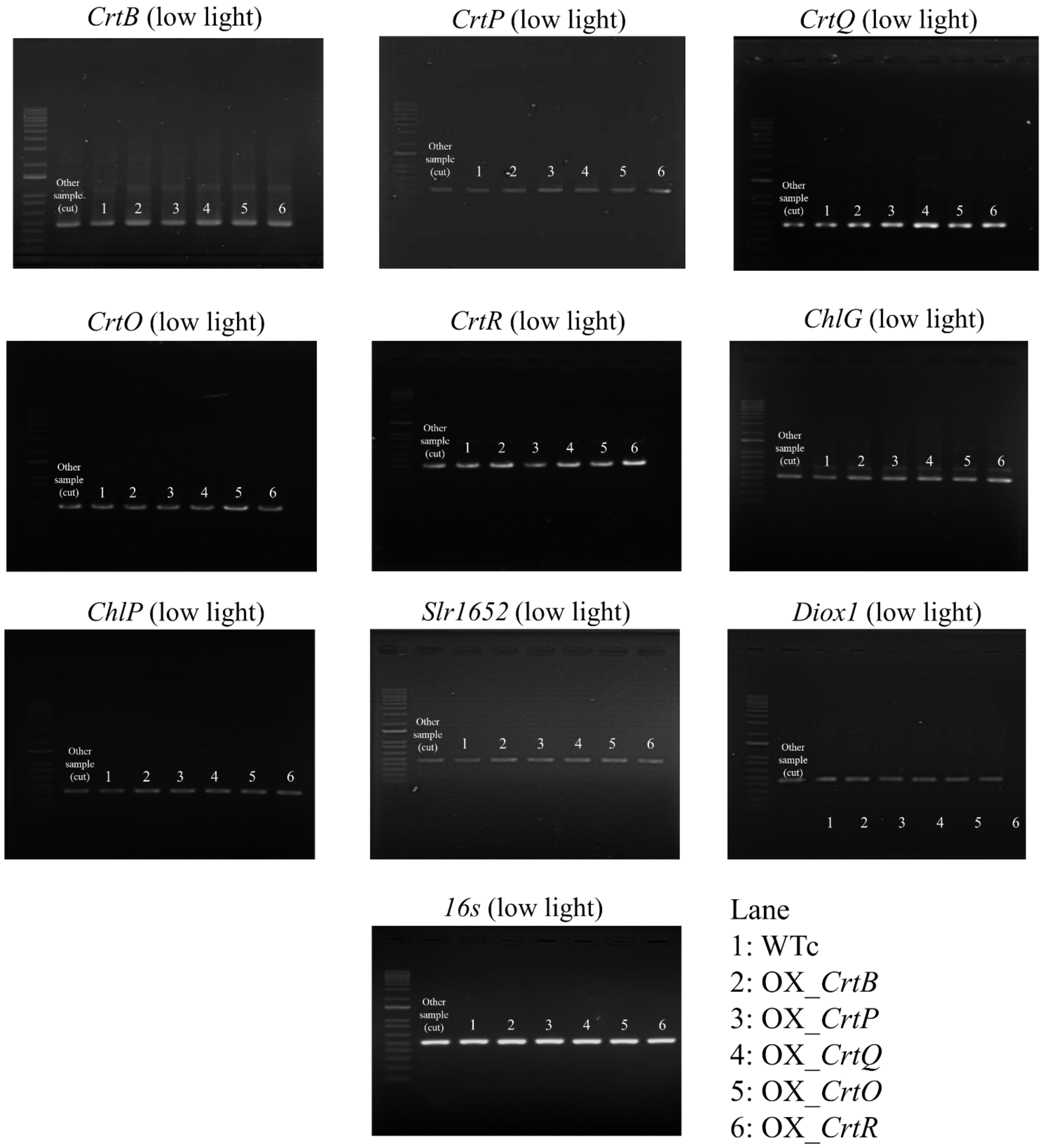

Supplementary Information, Figure S4

Agarose gel electrophoresis of RT-PCR product of carotenoids biosynthetic genes and related gene transcripts in all of *Synechocystis* sp. PCC 6803 strains grown under high light condition. The *16s* rRNA transcript was used as the reference.

CrtB (high light)

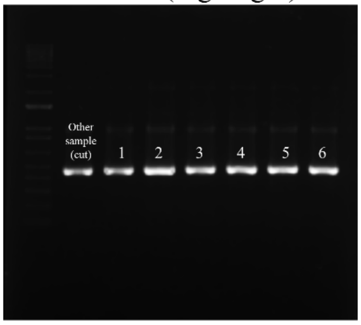

CrtP (high light)

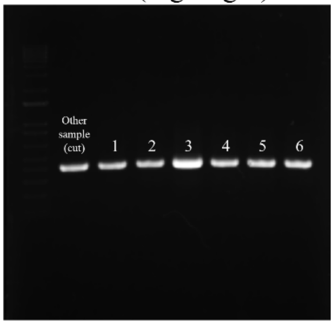

CrtQ (high light)

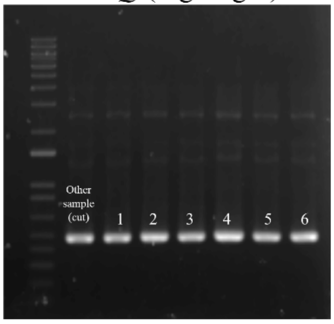

CrtO (high light)

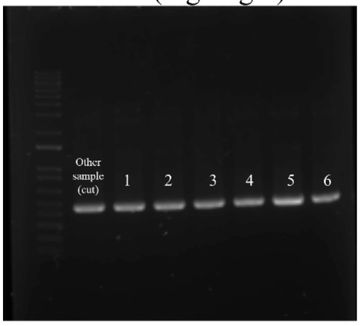

CrtR (high light)

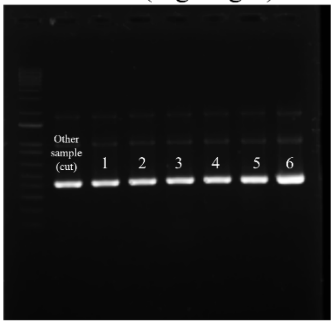

ChlG (high light)

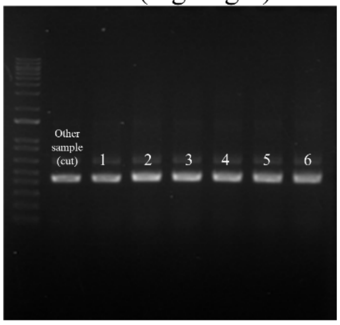

ChlP (high light)

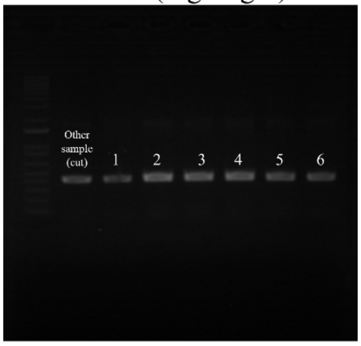

Slr1652 (high light)

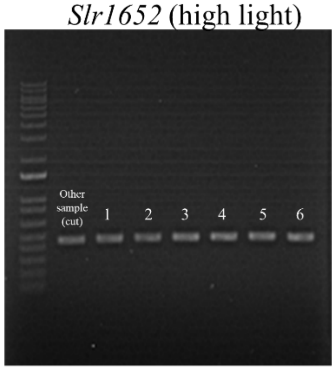

Diox1 (high light)

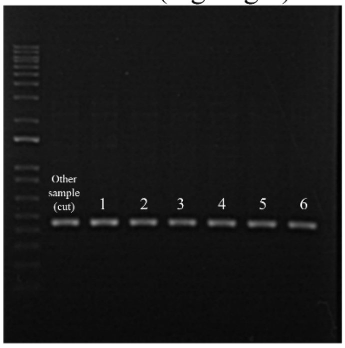

16S (high light)

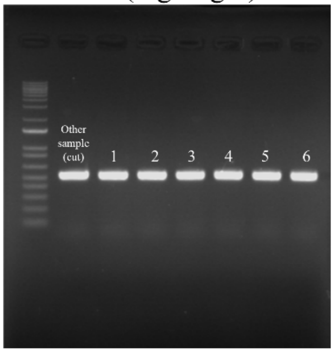

Lane

1: WTc

2: OX_*CrtB*

3: OX_*CrtP*

4: OX_*CrtQ*

5: OX_*CrtO*

6: OX_*CrtR*
